# Obesity, Adipocytokines and Cancer

**Published:** 2008-03-17

**Authors:** Takayuki Masaki, Hironobu Yoshimatsu

**Affiliations:** Department of Internal Medicine 1, Faculty of Medicine, Oita University, 1-1 Idaigaoka, Yufu-Hasama, Oita, 879-5593 Japan

**Keywords:** obesity, visceral adiposity, leptin, adiponectin, cancer

## Abstract

A great amount of literature has demonstrated a connection between obesity, visceral fat and the metabolic disorders such as hyperglycemia, hypertension, and hyperlipidemia. Lately, there has been an increased interest in understanding if cancer is related to obesity and visceral fat accumulation. The prevalence of both obesity and cancer are increasing and there has been keen interest in the relationship between visceral adiposity and the biology of cancers. White adipose tissue (WAT) provides a limitless capacity for triglyceride storage vital for survival. The concurrent rise in insulin, glucose, and lipids during meals stimulates triglyceride formation and storage in WAT. WAT is also recognized as an endocrine organ that secretes multiple cytokines such as leptin and adiponectin. In addition, leptin and adiponectin have been adipocytokines that attracted attention for cancer research. Thus, in this review, we will describe recent progress made in obesity, visceral adiposity, leptin and adiponectin in the involvement of various cancers.

## Introduction

Obesity is associated with significant morbidity and mortality and is increasing in prevalence worldwide. Weight gain is a major predictor of the metabolic syndrome with waist circumference being a more sensitive indicator as it reflects both subcutaneous and visceral adipose tissue (Matsuzawa Y, 2005). Associated conditions include type 2 diabetes, hypertension and dyslipidemia; a clustering of these has recently been termed as metabolic syndrome (Matsuzawa Y, 2005; Spiegelman BM et al. 2001; Friedman JM et al. 2000). Individuals affected with metabolic syndrome are at high risk factor for atherosclerosis and the resultant cardiovascular disease (Matsuzawa Y, 2006). The molecular mechanisms that underlie obesity and metabolic syndrome have not been fully elucidated, and effective therapeutic approaches are currently of general interest. The adipose tissue is also recognized as an endocrine organ that secretes multiple cytokines (Kershaw et al. 2004). Interestingly, visceral obesity and the related metabolic disorder have been identified as risk factors for several cancers, and associated with late-stage disease and a poor prognosis (Carroll KK et al. 1998; Bergstrom A et al. 2001; Bray GA, 2004; Vona-Davis L et al. 2007). Components of the metabolic syndrome, including visceral adiposity, insulin resistance, hyperglycemia and hyperinsulinemia have all been related to increased several cancer risk (Carroll KK et al. 1998; Bergstrom A et al. 2001; Bray GA, 2004; Vona-Davis L et al. 2007). Obesity, type 2 diabetes and the metabolic syndrome also have in common an increased production of leptin (Zhang et al. 1994; Kershaw et al. 2004) and a decreased production of adiponectin (Scherer PE et al. 1995; Hu E et al. 1996; Maeda K et al. 1996) by adipose tissue, with consequent elevations and reductions, respectively, in the circulating levels of these adipokines.

Potential roles of these adipocytokines in the regulation of cancer cell proliferation have been identified (Housa D et al. 2005; Garofalo C et al. 2006). Accumulating evidences suggest that leptin and adiponectin may have an important role in regulating the cancer growth and proliferation (Housa D et al. 2005; Garofalo C et al. 2006). Leptin and adiponectin act on cancer cells directly or indirectly by its receptors and downstream signaling pathways. These changes in plasma leptin and adiponectin, acting through endocrine and paracrine mechanisms, have been associated with an increase in cancer risk. Several studies showed that leptin stimulates, and adiponectin inhibits, tumor cell proliferation and the micro vessel angiogenesis which is essential for cancer development and progression (Vona-Davis L et al. 2007). Clinical studies also assess leptin and adiponectin levels in relation to risk from cancer, as well as mechanistic studies to prove these adipocytokines role in the development of cancer. Circulating levels of leptin and adiponectin is correlated with the risk of various cancers include breast, endometrial, colon/gastric and prostate cancers (Yang WS et al. 2002; Petridou E et al. 2003; Miyoshi Y et al. 2003). In this paper, we review the relationships between breast, endometrial, colon/gastric, prostate cancers and obesity.

## Obesity and Leptin

Adipocytokine leptin, a 16-kilodaltons-peptide hormone, was discovered through the identification of a mutation that produced obesity in *ob*/*ob* mice and involved in food intake and energy expenditure (Zhang Y et al. 1994). Leptin is mainly present in adipose tissue and blood and its receptors are abundant in peripheral tissues (Friedman JM, 1998). Circulating leptin levels parallel adipose tissue mass and reflect nutritional status because they decrease after fasting (Frederich RC et al. 1995; Saladin PE et al. 1995). In common with many hormonal signals, leptin has both circadian rhythmicity and short-term pulsatility in circulating levels (Ahima RS, 2006). Leptin exerts its biologic actions through the activation of its cognate receptors, which are encoded by the diabetes gene and belong to the class I cytokine receptor superfamily and whose signal transduction involves JAK/STAT pathways and adenosine monophosphate—activated protein kinase (Lago et al. 2007).

Circulating levels of leptin and its mRNA levels in adipose tissue are positively correlated with body mass index (Maffei M et al. 1995). In fact, levels of leptin and its mRNA have been shown to be higher in obese models (Maffei M et al. 1995). It has been suggested that high leptin levels are able themselves to induce leptin resistance via an increase in the levels of suppressor of cytokine signaling 3, a blocker of leptin receptor signaling (Enriori et al. 2006). At any rate, the state of leptin resistance in common human obesity remains to be completely elucidated, and further studies in this field are awaited. In anyway, these results indicated that a regulation of leptin and its receptors might be involved in the development of obesity or obesity-related metabolic disorders.

## Obesity and Adiponectin

Adiponectin is a 244—amino acid protein with structural homology to collagens VIII and X and complement factor C1q that is synthesized by adipose tissue and that increases fatty acid oxidation and reduces the synthesis of glucose in the liver and other tissues (Kadowaki and Yamauchi, 2005). Adiponectin has been identified in adipose tissue as a result of screening for adipose-specific genes (Scherer PE et al. 1995; Hu E et al. 1996; Maeda K et al. 1996). Mouse cDNAs for adiponectin, termed Acrp30 (Scherer PE et al. 1995) and AdipoQ (Hu E et al. 1996), were cloned by differential display before and after differentiation of mouse 3T3-L1 and 3T3-F442A cells. Adiponectin is present exclusively in adipose tissue and blood (Scherer PE et al. 1995; Hu E et al. 1996; Maeda K et al. 1996), and its receptors are abundant in peripheral tissues (Yamauchi T et al. 2003).

Circulating levels of adiponectin and its mRNA levels in adipose tissue are negatively correlated with body mass index (Arita Y et al. 1999). In fact, levels of adiponectin and its mRNA have been shown to be lower in obesity (Arita Y et al. 1999). In addition, patients with type 2 diabetes also showed lower levels of plasma adiponectin, indicating the involvement of this adipocytokine in glucose metabolism (Hotta K et al. 2000). Adiponectin treatment has been shown to improve insulin resistance (Berg H et al. 2001; Yamauchi T et al. 2001). Furthermore, adiponectin treatment has been shown to increase free fatty acid beta-oxidation via AMPK—dependent pathway (Yamauchi T et al. 2002), and adiponectin-deficient mice developed insulin resistance (Kubota N et al. 2002; Maeda N et al. 2002). Adiponectin interacts with two receptors (Adi-poR1 and AdipoR2) (Yamauchi T et al. 2003; Kadowaki T et al. 2005). AdipoR1 is expressed mainly in skeletal muscles while AdipoR2 is expressed mainly in the liver. Both AdipoR1 and AdipoR2 contain—G-protein coupled receptors—seven transmembrane domains (Yamauchi T et al. 2003; Kadowaki T et al. 2006). These findings suggest that a regulation of adiponectin and its receptors may be involved in the development of obesity or obesity-related metabolic disorders as well as leptin.

## Breast Cancer, Leptin and Adiponectin

Adipocytokine leptin is associated with various cancers including breast, endometrial, gastric, and prostate cancers (Tables 1, 2). Especially, circulating leptin concentrations are correlated with the incidence of breast carcinoma. Expression of leptin and its receptors were found in normal mammary tissue, as well as breast cancer tissue (O’Brien et al. 1999; Ishikawa et al. 2004). In addition, both leptin and its receptors have been elevated in cancer tissue compared with normal tissue (Ishikawa et al. 2004). Leptin induced the breast cancer carcinogenesis and invasion. The biological effect of leptin on breast cancer came from the findings of leptin-induced proliferation of breast cancer cells (Hu et al. 2002; Yin et al. 2004). Leptin exerts its growth effect on breast cancer cell lines through activation of multiple signaling cascades, including the JAK/STAT, ERK1/2, PKC- alpha and PPAR signaling pathways (Hu et al. 2002; Okumura et al. 2002; Garofalo et al. 2004; Yin et al. 2004). Leptin may influence breast cancer development in relation to ER status (Ray A et al. 2007). Gonzalez RR et al. demonstrated that the inhibition of leptin signaling could serve as a potential adjuvant therapy for treatment of breast cancer and provide a target for the designing strategies to prevent mammary tumor development (Gonzalez RR et al. 2007). Several clinical studies also demonstrated the relationship between obesity, leptin and breast cancer. However, the clinical studies on circulating leptin levels and breast cancer are not conclusive. Previous studies demonstrated that high body mass index and central obesity are associated with an increased risk of breast cancer (Chang et al. 1998; Harvie et al. 2003). Circulating leptin and leptin mRNA expression levels were both elevated in breast cancer patients and increase in leptin mRNA expression levels (Tessitore et al. 2000). Contrary, in breast cancer in situ, leptin does not appear to increase the risk of pre-menopausal cancer (Mantzoros et al. 1999). Sauter et al. also indicated serum levels of leptin were not associated with premenopausal or postmenopausal breast cancer (Sauter et al. 2004). Other studies demonstrated leptin are inversely related to breast cancer risk among premenopausal women (Petridou et al. 2000).

Low serum adiponectin is associated with high incidence of obesity-related cancer diseases, including breast cancers (Table 3.). Breast cancer cells expressed AdipoR1 and R2, but not adiponectin (Takahata C et al. 2007; Kornar A et al. 2007). Expression of AdipoR1, but not AdipoR2, was higher in breast tumor tissue than control tissues (Kornar A et al. 2007). Prolonged adiponectin treatment caused cell growth arrest and apoptosis of MCF7 and MDA-MB-231cell (Kang JH et al, 2005; Dieudonne et al, 2006). Exposure of T47D cells to high-molecular-weight adiponectin significantly inhibited the percentage of viable cells and proliferation (Kornar A et al. 2007). These effects were associated with activation of ERK1/2 but not AMP-activated protein kinase or p38MAPK (Kornar A et al. 2007). It is indicated that adiponectin can directly control cancer cell growth. Clinical data from different ethnic populations indicate that low adiponectin plasma level is an independent risk factor for breast cancer disease. An inverse relationship of circulating adiponectin levels and breast cancer risk in postmenopausal women was observed independently of possible effects of IGF-1, leptin, and BMI (Mantzoros et al. 2004). Miyoshi et al. also demonstrated that an association of low serum adiponectin levels with increased risk of breast cancer in both postmenopausal and premenopausal women (Miyoshi et al. 2003). Korner A et al. described that women with the lowest adiponectin levels had an increased risk of breast cancer (Korner A et al. 2007).

## Endometrial Cancer, Leptin and Adiponectin

Similar to breast cancer, leptin and adiponectin are associated with endometrial cancer. Leptin receptor mRNA and its proteins are expressed in the endometrium (Koda et al. 2007). The effect of leptin on endometrial cancer carcinogenesis is due to leptin-induced cell growth of endometrial cancer. Leptin promotes endometrial cancer growth and invasiveness and implicate the JAK/STAT and AKT pathways (Sharma D et al. 2006). Leptin could exert autocrine effect to stimulate endometrial cancer progression (Koda et al. 2007). The relationship between obesity, leptin and endometrial cancer risk have been observed in clinical studies. Obesity is strongly related to endometrial cancer (Calle EE and Thun MJ, 2004). A linear increase in the risk of endometrial cancer with increasing body weight has been observed (Calle EE et al. 2003). Circulating leptin levels were positively associated with endometrial cancer (Petridou et al. 2002). Similarly, Yuan et al. showed that circulating leptin was higher in endometrial cancer patients, however this association was not observed after BMI normalization (Yuan et al. 2004).

Adiponectin receptors were expressed in the human endometrium (Takemura Y et al. 2006). Cong et al. demonstrated that adiponectin leads to suppression of cell proliferation in human endometrial carcinoma cell, which is primarily due to the significant increase of cell populations at G (1)/G (0)-phase and to the induction of apoptosis (Cong et al. 2007). It is suggested that adiponectin exerts direct anti-proliferative effects on the cells by inducing cell cycle arrest and apoptosis (Cong et al. 2007). An inverse association of endometrial cancer in women and adiponectin had independent roles in promoting endometrial cancer (Petridou et al. 2003; Dal Maso et al. 2004). High circulating adiponectin levels are associated with reduced endometrial cancer risk, largely independent of other obesity-related risk factors (Cust AE et al. 2007). The association remained statistically significant after separate adjustment for other obesity-related physiological risk factors such as C-peptide (Cust AE et al. 2007).

## Colon/gastric Cancer, Leptin and Adiponectin

Colon cancer is the common cause of cancer death and the rapid increase in the cancer incidence in several populations suggest environmental factors, including high-fat diet and obesity (Frezza EE et al. 2006). High fat diet that increase circulating leptin promote carcinogenesis by stimulating colon cell proliferation (Liu Z et al. 2001). Leptin receptors were expressed in both tumor tissue and colon cancer cell line HT29 (Hardwick JC et al. 2001). Leptin induced cell proliferation and p42/44 MAPK phosphorylation (Hardwick JC et al. 2001). Previous study showed that leptin promotes invasiveness of colonic epithelial cells via phosphoinositide 3-kinase-, rho-, and rac-dependent signaling pathways (Attoub et al. 2000). Leptin was shown to work as a mitogen for intestinal epithelial cells and also decreased apoptotic cell death in a cancer cell line (Rouet-Benzineb P et al. 2004). Leptin has also been shown to enhance the development of adenomatous lesions in genetically obese mice (Hirose Y et al. 2004). In contrast, a study showed that leptin did not promote the growth of colon cancer xenografts in nude mice (Aparicio T et al. 2005). Obesity has been associated with higher risk of colorectal cancer and adenomas in men and women (Calle and Thun, 2004; Giovannucci et al. 1995). The link between colorectal cancer and obesity is more significant in men than in women (Terry et al. 2001, 2002; Stattin et al. 2003). On the other hand, no difference was found between serum leptin levels in cancer patients and controls (Tessitore et al. 2000). Other authors demonstrated that circulating leptin levels in patients with colon cancer were significantly decreased (Arpaci et al. 2002). In the study, leptin levels were lower in colon cancer patients despite lack of weight loss and BMI measurements comparable to that of control subjects. Thus, further studies would be necessary for clarify the relationship leptin and colon cancer in clinical study. Adiponectin potently induced apoptosis and inhibited the proliferation of AZ521 cells (Ishikawa et al. 2007). Down-regulation of either AdipoR1 or AdipoR2 by specific siRNA significantly suppressed the growth inhibitory effects of adiponectin in the cell line. In addition, adiponectin negatively regulates the progression of gastric cancer cells possibly through both AdipoR1 and AdipoR2. Circulating adiponectin levels are inversely correlated with the risk of colorectal cancer (Ferroni et al. 2007). The median adiponectin levels were lower in colorectal cancer patients than controls (Ferroni et al. 2007). Moreover, median adiponectin concentration gradually decreased with increase in tumor stage. Thus, low serum adiponectin might represent an adjunctive tool in risk prediction for colorectal cancer recurrence.

## Prostate Cancer, Leptin and Adiponectin

Although several data appeared conflicting, recent studies have clarified the association between leptin and prostate cancer. Leptin induced *in vitro* proliferation and inhibited apoptosis of prostate cell lines Somasundar et al. (2004). The effects of leptin were mediated through activation of a short form of leptin receptor. In addition, the activation of PI3 and MAPK pathway were shown to be involved in the process of leptin-induced proliferation. Clinical studies focused on comparison of serum leptin levels in benign prostatic hyperplasia, prostate cancer and control healthy subjects (Schuurman et al. 2000; Jonsson et al. 2003; Hubbard et al. 2004). The studies on circulating leptin levels and prostate cancer are not conclusive. Moderately elevated plasma leptin concentrations are associated with later development of prostate cancer (Stattin et al. 2001; Saglam et al. 2003). Single nucleotide polymorphism G/A in region—2548 bp of leptin gene was associated with the risk of prostate cancer development and more advanced stage in already developed cancer (Ribeiro et al. 2004). By contrast, the association between leptin levels and prostate cancer risk was not significant (Lagiou P et al. 1998; Stattin et al. 2003). As well as the above cancers, further studies would be necessary for clarify the relationship leptin and prostate cancer in clinical study. In prostate cancer cells, adiponectin inhibited the cell growth (Bub JD et al. 2006). Adiponectin can activate AMPK in cell, suggesting that adiponectin may suppress tumor development through AMPK activation and subsequent inhibition of mammalian target of rapamycin (mTOR) (Barb D et al. 2007). This adiponectin stimulation of mTOR was mediated through phosphatidylinositol 3-kinase and Akt activation (Barb D et al. 2007). These results show that adiponectin can activate both AMPK and PI3 kinase/Akt pathways, and that cell type-specific factors may determine which of these pathways will have the dominant effect on mTOR.

## Conclusions

It has been described that obesity, leptin and adiponectin are related with the risk of some types of cancer such as that of the breast, endometrium, colon and prostate. In these cancer cell lines, adipocytokine leptin stimulates cell growth, proliferation and invasion of the cells in vitro. Contrary, adiponectin down-regulates the cancer cell growth and proliferation. Evidence suggests a role for obesity, leptin and adiponectin in regulating the progression of established cancer. Emerging data suggest obesity increases the risk of aggressive cancer, while simultaneously decreasing the risk of more indolent disease. This is likely driven by both “biological” and “nonbiological” causes. Despite several biological mechanisms that potentially link obesity to cancer, the effects of obesity on the subsequent effects on the detection of cancer, are not consistent according to the previous literature. Additionally, the epidemiologic data for the incidence of cancer in obese and non-obese populations are conflicting. It is difficult to determine whether oncologic and functional outcomes in obese patients differ substantially from those in non-obese patients. Hopefully, the increasing focus on these health problems might further elucidate their complex relationship in the future.

## Figures and Tables

**Table 1:** Effect or association of leptin on breast and endometrial cancer.

Type of cancer	Effect or association	References
Breast	Increased cell growth and proliferation	(Hu et al. 2002; Yin et al. 2004)
	Activation of JAK/STAT and ERK 1/2 signaling	(Hu et al. 2002; Yin et al. 2004)
	Relation to ER status	(Ray et al. 2007)
	Potential adjuvant therapy	(Gonzalez et al. 2007)
	Positive association with breast cancer	(Tessitore et al. 2000)
	Lack of association with cancer risk	(Mantzoros et al. 1999; Sauter et al. 2004)
	Negative association with cancer risk	(Petridou et al. 2000)
Endometrium	Activation of the JAK/STAT signaling	(Sharma et al. 2006)
	Autocrine effect to stimulate cancer	(Koda et al. 2006)
	Positive association with endometrial cancer	(Petridou et al. 2002)

**Table 2:** Effect or association of leptin on colonal and prostate cancer.

Type of cancer	Effect or association	References
Colon	Promoted invasiveness by the PI-3K, Rhoand rac signaling	(Attoub et al. 2000)
	Increased colonic cell growth	(Hardwick et al. 2001)
	Positive association with cancer risk	(Stattin et al. 2003)
	Lack of association with cancer risk	(Tessitore et al. 2000)
Prostate	Increased cell proliferation and suppression of apoptosis	(Somasunder et al. 2004)
	Positive association with cancer risk	(Stattin et al. 2001; Saglam et al. 2003)
	Lack of association with cancer risk	(Lagiou et al. 1998; Stattin et al. 2003)

**Table 3:** Effect or association of adiponectin on cancer.

Type of cancer	Effect or association	References
Breast	Regulate cell growth, and proliferative response	(Kang et al. 2005; Dieudonne et al. 2006; Korner et al. 2007).
	Inverse relationship of adiponectin levels and the cancer risk	(Mantrozos et al. 2004; Miyoshi et al. 2003; Korner et al. 2007)
Endometrial	Regulate cell growth, and proliferative response	(Cong et al. 2007)
	Inverse relationship of adiponectin levels and the cancer risk	(Petridou et al. 2003; Dal Maso et al. 2004; Cust et al. 2007)
Colon	Inverse relationship	(Ferroni et al. 2007)
Gastric	Regulate proliferative response	(Ishikawa et al. 2007)
Prostate	Regulate cell growth	(Bub et al. 2006; Barb et al. 2007)
